# Vitamin D Status and Gastroenteropancreatic Neuroendocrine Neoplasms: Biological Rationale, Clinical Associations and Limitations of Current Evidence

**DOI:** 10.3390/cancers18142346

**Published:** 2026-07-20

**Authors:** Bartosz Basiaga, Violetta Rosiek, Beata Kos-Kudła

**Affiliations:** Department of Endocrinology and Neuroendocrine Tumors, ENETS Centre of Excellence, Department of Pathophysiology and Endocrinology, Medical University of Silesia, 40-514 Katowice, Poland

**Keywords:** gastroenteropancreatic neuroendocrine neoplasms, GEP-NEN, neuroendocrine tumors, vitamin D, 25-hydroxyvitamin D, vitamin D receptor, Ki-67, bone metabolism, malabsorption, prognosis

## Abstract

Background: Vitamin D deficiency is common in patients with gastroenteropancreatic neuroendocrine neoplasms (GEP-NENs). Whether vitamin D status influences tumor aggressiveness and clinical outcomes remains uncertain. This review examines current evidence on the prevalence, determinants, clinical associations, and clinical relevance of vitamin D deficiency in GEP-NENs. Methods: A narrative and critical review of the literature was conducted using the PubMed/MEDLINE, Scopus, and Web of Science databases. Clinical studies, observational cohorts, translational research, selected mechanistic studies, and current clinical guidelines addressing vitamin D metabolism and neuroendocrine neoplasms were evaluated. Results: Vitamin D deficiency has been reported in approximately 60–80% of patients with GEP-NENs. Low serum 25-hydroxyvitamin D concentrations likely reflect multiple disease-related factors, including malabsorption, pancreatic exocrine insufficiency, chronic diarrhea, previous gastrointestinal surgery, and nutritional impairment. Several observational studies have linked lower vitamin D status with markers of more aggressive disease, including higher Ki-67 proliferation index values and shorter progression-free survival. Nevertheless, the available data are heterogeneous, predominantly observational, and do not support a causal relationship between vitamin D deficiency and tumor progression. At present, the main clinical rationale for assessing vitamin D status is to support metabolic care, preserve bone health, and prevent osteoporosis. Conclusions: Vitamin D deficiency is a frequent and clinically relevant comorbidity in patients with GEP-NENs. Although lower vitamin D status has been associated with markers of more aggressive disease, current findings do not support vitamin D supplementation as an anticancer treatment strategy. Prospective clinical and translational studies are needed to clarify the biological and clinical significance of vitamin D signaling in GEP-NENs.

## Simple Summary

Vitamin D deficiency is common in patients with gastroenteropancreatic neuroendocrine neoplasms (GEP-NENs), largely because of disease-related factors such as malabsorption, pancreatic exocrine insufficiency, chronic diarrhea, nutritional impairment, and previous gastrointestinal surgery. Some observational studies have associated low vitamin D levels with features of more aggressive disease, although a causal relationship has not been established. This review summarizes current knowledge on vitamin D deficiency in GEP-NENs and highlights its clinical relevance, particularly for bone health and supportive care, rather than as an anticancer treatment strategy.

## 1. Introduction

Neuroendocrine neoplasms (NENs) are a heterogeneous group of tumors arising from diffuse neuroendocrine cells capable of producing hormones and biologically active peptides. Gastroenteropancreatic neuroendocrine neoplasms (GEP-NENs) represent the most common subgroup and account for the majority of clinically relevant neuroendocrine tumors [1,2].

The incidence of NENs has increased steadily over recent decades, partly because of improved diagnostic techniques and increased disease recognition, although population-based studies also suggest a true rise in disease occurrence [3,4]. Consequently, an increasing number of patients require long-term multidisciplinary management [3].

GEP-NENs comprise the largest subgroup of NENs and display marked biological heterogeneity, ranging from indolent tumors to highly aggressive neoplasms with distinct prognostic and therapeutic implications [2,5].

According to the current World Health Organization (WHO) classification, NENs are categorized as well-differentiated neuroendocrine tumors (NETs) or poorly differentiated neuroendocrine carcinomas (NECs), with histological differentiation and Ki-67 proliferation index representing the principal prognostic factors [2,6,7].

Despite advances in diagnosis and treatment, many patients are still diagnosed with advanced disease, and increasing attention has been directed toward metabolic and nutritional complications that seem to affect long-term outcomes and quality of life [2,8,9,10,11,12,13,14].

Vitamin D deficiency is among the most frequently observed metabolic abnormalities in patients with GEP-NENs. Vitamin D plays an essential role in calcium-phosphate homeostasis and bone metabolism, but it is also involved in the regulation of cell proliferation, differentiation, apoptosis, and immune response [15,16]. Many of these effects are mediated through the vitamin D receptor (VDR), a ligand-dependent transcription factor regulating genes associated with cellular homeostasis and carcinogenesis [17]. The presence of VDR in extraskeletal tissues has increased interest in the potential role of vitamin D signaling in cancer biology [18,19].

Several disease-related factors may contribute to vitamin D deficiency in patients with GEP-NENs, including chronic diarrhea, malabsorption, pancreatic exocrine insufficiency, and previous gastrointestinal surgery [20]. Observational studies have reported associations between low serum 25-hydroxyvitamin D concentrations and markers of more aggressive disease, including higher Ki-67 index values, higher tumor grade, and shorter progression-free survival [21,22,23]. However, the available evidence remains heterogeneous and predominantly observational. Interpretation of these findings is further limited by the biological heterogeneity of GEP-NENs, differences between study populations, and multiple potential confounding factors. Current data therefore do not support conclusions regarding causality.

Particular attention should also be given to hereditary syndromes associated with NEN development, especially multiple endocrine neoplasia type 1 (MEN1), but also multiple endocrine neoplasia type 2 (MEN2), von Hippel–Lindau disease, tuberous sclerosis complex, and neurofibromatosis type 1 [24,25]. In MEN1, coexisting primary hyperparathyroidism and chronic disturbances in calcium-phosphate metabolism may further complicate both the interpretation and clinical consequences of vitamin D deficiency [26].

The aim of this review is to critically summarize current data regarding vitamin D status in patients with GEP-NENs, with particular emphasis on the biological background, prevalence and determinants of vitamin D deficiency, reported associations with tumor aggressiveness, and clinical implications, particularly in the context of bone health. Throughout this review, the term “vitamin D” refers to clinically assessed or mechanistically discussed vitamin D metabolites, including 25(OH)D and 1,25(OH)_2_D_3_ where appropriate.

Although the primary focus of this review is GEP-NENs, selected findings derived from mixed neuroendocrine neoplasm cohorts and non-NEN experimental cancer models are discussed separately when considered biologically or clinically relevant.

## 2. Materials and Methods

In this review, the term GEP-NENs is used as the overarching term for gastroenteropancreatic neuroendocrine neoplasms. The term GEP-NETs is used only when referring specifically to well-differentiated neuroendocrine tumors or when this terminology was used in the cited studies. The review focused on the biological background of vitamin D regulation, determinants of vitamin D deficiency, clinical associations, prognostic implications, bone health, and supportive metabolic care in patients with GEP-NENs. This review was designed as a narrative and critical review rather than a systematic review or meta-analysis. Therefore, no formal PRISMA-based study selection process or quantitative synthesis was performed.

### 2.1. Literature Search and Study Selection

**Figure 1:**
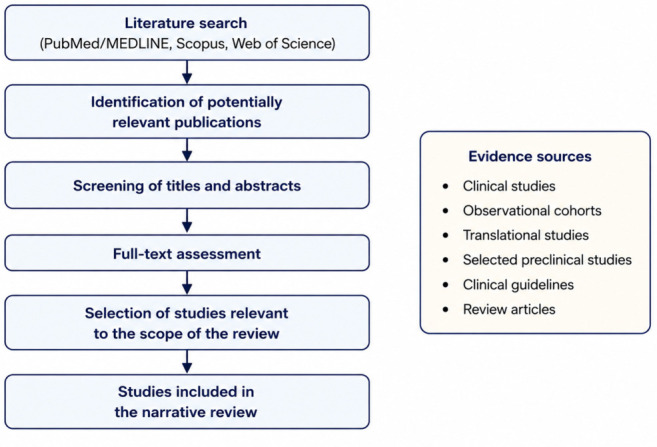
Flowchart illustrating the literature search and study selection process used in this narrative review. Studies were identified through searches of the PubMed/MEDLINE, Scopus, and Web of Science databases, followed by title and abstract screening, full-text assessment, and selection of studies relevant to the scope of the review.

A literature search was performed using the PubMed/MEDLINE, Scopus, and Web of Science databases. The final search was conducted in January 2026. Predefined keyword combinations and Medical Subject Headings (MeSH) related to vitamin D metabolism and neuroendocrine neoplasms were used. The literature search and study selection process is illustrated in Figure 1.

The search strategy included terms such as: (“vitamin D” or “25-hydroxyvitamin D” or “1,25-dihydroxyvitamin D” or “vitamin D receptor” or “VDR”) and (“gastroenteropancreatic neuroendocrine neoplasms” or “GEP-NEN” or “GEP-NET” or “neuroendocrine tumors” or “neuroendocrine neoplasms” or “pancreatic neuroendocrine tumors” or “gastrointestinal neuroendocrine tumors”).

Reference lists of selected articles and review papers were also screened to identify additional relevant studies.

After duplicate removal, titles and abstracts were screened for relevance. Full-text evaluation was then performed for studies considered relevant to the scope of the review. Priority was given to studies focused directly on GEP-NEN populations. However, selected data from mixed NET/NEN cohorts and non-NEN experimental cancer models were also included when they provided important biological or clinical context. The literature search strategy is summarized in Table 1.

**Table 1:** Summary of literature search strategy.

Database	Search Terms	Time Range	Main Focus
PubMed/MEDLINE	Representative vitamin D- and GEP-NEN-related search terms	inception–January 2026	Clinical and translational studies
Scopus	Representative vitamin D- and GEP-NEN-related search terms	inception–January 2026	Observational and mechanistic studies
Web of science	Representative vitamin D- and GEP-NEN-related search terms	inception–January 2026	Clinical reviews and translational evidence

Abbreviations: GEP-NEN, gastroenteropancreatic neuroendocrine neoplasm.

### 2.2. Inclusion and Exclusion Criteria

The review included original clinical studies, observational cohorts, case–control studies, translational research, selected preclinical studies, review articles, and current clinical guidelines related to vitamin D metabolism and neuroendocrine neoplasms. Studies addressing bone metabolism, malabsorption, and supportive metabolic care were also included.

Particular attention was given to studies evaluating serum 25-hydroxyvitamin D concentrations, Ki-67 proliferation index, tumor grade, disease progression, progression-free survival (PFS), overall survival (OS), bone health, nutritional impairment, and metabolic complications in patients with GEP-NENs.

Studies focused directly on GEP-NENs were prioritized whenever possible. However, because of the limited number of available studies, selected data from mixed NET/NEN cohorts and mechanistic studies from other cancer models were also included when considered biologically or clinically relevant. These findings were interpreted separately from direct GEP-NEN evidence.

Case reports with limited clinical relevance, studies without adequate tumor characterization, publications lacking clear distinction between GEP-NENs and other malignancies, and studies without clearly described vitamin D assessment methods were excluded from the primary analysis.

### 2.3. Evidence Synthesis Approach

To reduce overinterpretation of the available data, evidence was stratified according to study type and patient population. Clinical findings derived directly from GEP-NEN cohorts were distinguished from studies involving mixed NET/NEN populations and from mechanistic findings based on non-NEN experimental models.

The review focused mainly on observational clinical studies evaluating serum 25(OH)D concentrations, tumor grade, Ki-67 proliferation index, progression-free survival, overall survival, bone metabolism, malabsorption, and nutritional status in patients with GEP-NENs.

Findings derived from experimental and translational models were interpreted cautiously and used primarily to provide biological context. Quantitative meta-analysis was not performed because of substantial heterogeneity between studies, including differences in patient populations, laboratory methods, definitions of vitamin D deficiency, and study design.

### 2.4. Methodological Considerations and Limitations

Most available data regarding vitamin D status in GEP-NENs are derived from observational studies with relatively small and heterogeneous patient cohorts. Important limitations of the current literature include variability in definitions of vitamin D deficiency, differences in laboratory assessment methods, seasonal variation in 25(OH)D measurements, and the influence of confounding factors such as disease stage, nutritional status, malabsorption, systemic treatment, and concomitant metabolic disorders.

In addition, several studies included mixed NET/NEN populations rather than exclusively GEP-NEN cohorts, which limits direct comparison between studies. Since most currently available data continue to be observational, reported associations between vitamin D status and tumor behavior should not be interpreted as evidence of causality.

Comparative tables were prepared to summarize the most clinically and biologically relevant findings discussed in this review.

## 3. Biology of Vitamin D Metabolism and Signaling in Cancer

### 3.1. Vitamin D Metabolism, Classical and Non-Canonical Pathways, and Potential Relevance in Cancer

Vitamin D exists primarily as vitamin D_3_ (cholecalciferol) and vitamin D_2_ (ergocalciferol). Vitamin D_3_ is synthesized in the skin following ultraviolet B (UVB) exposure, whereas dietary intake and supplementation become increasingly relevant in patients with chronic diseases [27]. After sequential hydroxylation, vitamin D is converted in the liver to 25-hydroxyvitamin D (25(OH)D), mainly by CYP2R1, and subsequently in the kidneys by CYP27B1 to its biologically active form, 1,25-dihydroxyvitamin D (1,25(OH)_2_D), the principal ligand of the VDR [27,28,29,30]. Because of its relatively long half-life and stable circulating concentration, serum 25(OH)D remains the standard marker of vitamin D status in clinical practice [30].

An important regulatory step is CYP24A1-mediated catabolism, which limits the activity of both 25(OH)D and 1,25(OH)_2_D. Increased CYP24A1 expression has been reported in several malignancies and seems to attenuate local VDR signaling, indicating that circulating 25(OH)D concentrations may not fully reflect vitamin D activity within the tumor microenvironment [31,32]. In addition, CYP27B1 expression in immune cells and selected tumor tissues supports the concept of local autocrine and paracrine vitamin D activation, although current data are derived predominantly from experimental studies [33,34,35].

An alternative CYP11A1-dependent pathway of vitamin D metabolism has also been described. It generates hydroxylated metabolites, including 20(OH)D and 20,23(OH)_2_D, which have demonstrated biological activity in preclinical models and may modulate VDR signaling with potentially lower calcemic effects than 1,25(OH)_2_D [36,37]. Selected non-canonical vitamin D metabolites also seem to also interact with retinoic acid-related orphan receptors (RORα and RORγ), linking vitamin D signaling to immune and inflammatory pathways implicated in cancer biology [36,37,38]. However, findings supporting these mechanisms are derived almost exclusively from experimental models, and their biological and clinical relevance in GEP-NENs remains uncertain.

### 3.2. Vitamin D Receptor, Genomic and Non-Genomic Mechanisms, and Epigenetic Regulation

**Figure 2:**
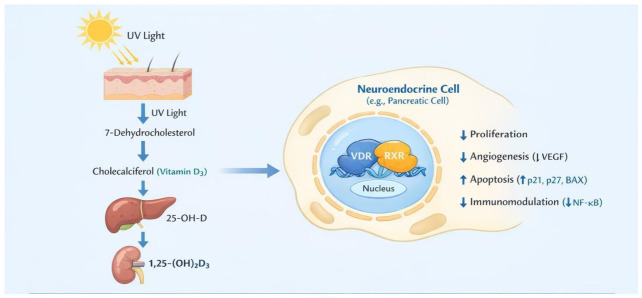
Overview of vitamin D metabolism and VDR signaling in cancer biology. Vitamin D synthesis begins in the skin under ultraviolet B (UVB) exposure, leading to the formation of cholecalciferol (vitamin D_3_). Subsequent hydroxylation in the liver produces 25-hydroxyvitamin D (25(OH)D), which is further converted in the kidneys and selected extra-renal tissues into the biologically active form, 1,25-dihydroxyvitamin D (1,25(OH)_2_D_3_). Active vitamin D binds to the vitamin D receptor (VDR), which forms a heterodimer with the retinoid X receptor (RXR) and regulates transcription of genes involved in cell cycle control, differentiation, apoptosis, immune regulation, and angiogenesis. Experimental studies suggest that vitamin D signaling may influence inflammatory pathways and mechanisms related to tumor progression and the tumor microenvironment [17,29,30,32,39]. Abbreviations: UVB, ultraviolet B; 25(OH)D, 25-hydroxyvitamin D; 1,25(OH)_2_D_3_, 1,25-dihydroxyvitamin D_3_; VDR, vitamin D receptor; RXR, retinoid X receptor; ↑ indicates increased activity or expressio; ↓ indicates decreased activity or expression.

The vitamin D receptor is a ligand-dependent transcription factor belonging to the nuclear receptor family. Following binding of 1,25(OH)_2_D, it forms a heterodimer with the retinoid X receptor (RXR) and interacts with vitamin D response elements to regulate genes involved in cell cycle control, apoptosis, differentiation, oxidative stress, and immune signaling [29,30,35]. Figure 2 summarizes the principal components of the vitamin D-VDR axis in cancer biology.

VDR signaling has been implicated in several processes relevant to cancer, including the regulation of cellular proliferation, apoptosis, angiogenesis, and interactions within the tumor microenvironment [30,32,40]. Altered VDR activity has been described in multiple malignancies and seems to result from changes in vitamin D metabolism, reduced receptor expression, or disturbed transcriptional regulation involving genes such as *CDKN1A* (*p21*) [32,39]. Most of these observations originate from preclinical research, and their biological significance in GEP-NENs remains unclear.

Vitamin D also exerts rapid non-genomic effects through secondary signaling pathways that influence ion transport, cell adhesion, migration, and other short-term cellular responses [41,42]. Cellular responsiveness to vitamin D appears to depend not only on ligand availability and VDR expression but also on epigenetic regulation, including chromatin accessibility and interactions with other transcription factors [43,44,45]. Although these mechanisms are increasingly recognized in cancer biology, they have not yet been directly demonstrated in GEP-NENs. Table 2 summarizes the major processes of VDR signaling discussed in cancer research.

### 3.3. The VDR Axis, ROR Receptors, and Immunomodulation in the Tumor Microenvironment

Vitamin D contributes to immune regulation through both systemic and local mechanisms. Immune cells, including macrophages and antigen-presenting cells, are thought to express CYP27B1, enabling local production of active vitamin D metabolites within inflammatory sites and the tumor microenvironment [33,34,35].

Vitamin D signaling has been shown to modulate inflammatory responses by influencing lymphocyte differentiation, dendritic cell function, and the expression of selected pro-inflammatory cytokines [33,34,47]. Because chronic inflammation contributes to carcinogenesis and tumor progression, these observations have strengthened interest in the role of vitamin D signaling in cancer biology [48].

Non-canonical vitamin D metabolites may also interact with retinoic acid-related orphan receptors (RORα and RORγ). Experimental studies suggest that selected metabolites may act as inverse agonists of these receptors, thereby influencing immune and inflammatory signaling pathways [36,37,38]. RORγt is a key regulator of Th17-cell differentiation, whereas IL-17 signaling has been linked to pro-inflammatory and proangiogenic activity in several experimental cancer models [46,49]. However, these findings originate predominantly from preclinical and non-NEN studies, and their relevance to GEP-NEN biology remains uncertain.

Taken together, the available evidence supports a biologically plausible role for vitamin D in immune regulation and tumor biology but not a clinically established contribution in GEP-NENs. Whether these pathways influence disease behavior or treatment response continues to be unknown, and prospective clinical studies addressing these mechanisms are still lacking [32,33,47].

Table 3 summarizes the principal vitamin D metabolic pathways and their proposed relevance to cancer biology, with particular emphasis on processes potentially related to neuroendocrine neoplasms.

### 3.4. Vitamin D Receptor Expression in Neuroendocrine Neoplasms

Direct evidence on VDR expression and activity in gastroenteropancreatic neuroendocrine neoplasms continues to be limited. Although VDR has been extensively studied in several solid tumors, including colorectal, breast, and prostate cancers, only a limited number of studies have examined its role in neuroendocrine neoplasms.

Although components of the vitamin D signaling pathway have been identified in neuroendocrine tissues, the prevalence, functional significance, and prognostic value of VDR expression in GEP-NENs remain poorly defined. This lack of tumor-specific data represents an important gap in current knowledge. Further translational studies evaluating VDR expression, receptor activity, and interactions with the tumor microenvironment are needed to determine whether vitamin D signaling influences GEP-NEN progression and clinical behavior.

At present, VDR-related mechanisms should be regarded as biologically plausible but insufficiently validated in GEP-NENs, and their clinical relevance remains uncertain.

## 4. Prevalence and Determinants of Vitamin D Deficiency in Patients with GEP-NEN

### 4.1. Prevalence of 25-Hydroxyvitamin D Deficiency in GEP-NENs

Available clinical data indicate that vitamin D deficiency is common in patients with GEP-NENs. Observational studies reported reduced serum 25(OH)D concentrations in a substantial proportion of patients, with the prevalence of vitamin D deficiency ranging from approximately 60% to 80%, depending on the study design, patient population, and diagnostic criteria applied [21,22,23].

In the study by Massironi et al., most patients with GEP-NENs had serum 25(OH)D concentrations below the recommended range, regardless of the primary tumor site [21]. Similar findings were reported in other European cohorts, where vitamin D deficiency was identified in nearly three-quarters of patients with well-differentiated neuroendocrine tumors [22,23].

The available GEP-NEN studies used broadly comparable definitions of vitamin D deficiency. Massironi et al. and Albertelli et al. defined deficiency as serum 25(OH)D concentrations ≤ 20 ng/mL, whereas Altieri et al. used a cut-off value of <20 ng/mL and additionally distinguished vitamin D insufficiency (20–30 ng/mL) from sufficient vitamin D status (>30 ng/mL). Therefore, differences in the reported prevalence of vitamin D deficiency are unlikely to be explained primarily by the cut-off values used but rather by differences in patient populations, disease characteristics, and study design [21,22,23].

The remaining variability between studies is more likely to reflect differences in patient populations, disease stage, treatment strategies, laboratory methods, and other methodological factors. Taken together, the available evidence indicates that vitamin D deficiency is highly prevalent in patients with GEP-NENs and may occur more frequently than in geographically comparable general populations. Disease-related and treatment-related factors probably contribute to this observation, although direct controlled comparisons remain limited.

### 4.2. Disease-Specific Factors Contributing to Vitamin D Deficiency in GEP-NENs

Several disease-related factors could contribute to vitamin D deficiency in patients with GEP-NENs. Intestinal malabsorption appears to play a major role, particularly in patients after small bowel or pancreatic resection, where absorption of fats and fat-soluble vitamins may be impaired [50,51,52,53].

Exocrine pancreatic insufficiency, commonly observed in pancreatic neuroendocrine tumors and after pancreatic surgery, could further contribute to chronic malabsorption and persistent vitamin D deficiency despite oral supplementation [51,53].

The potential contribution of somatostatin analogues (SSAs) has also been investigated. Current findings suggests that SSA treatment alone is unlikely to represent an independent cause of vitamin D deficiency. Reduced 25(OH)D concentrations probably reflect the combined effects of malabsorption, nutritional impairment, disease burden, and previous gastrointestinal interventions [52,54].

In one study evaluating fat-soluble vitamin status in patients with small intestinal NENs treated with SSAs for at least 18 months, approximately 80% of patients presented with deficiency of at least one fat-soluble vitamin, while nearly one-third had multiple deficiencies [55]. These findings support periodic nutritional assessment and monitoring of fat-soluble vitamin status in selected patients receiving long-term SSA therapy [55].

**Figure 3:**
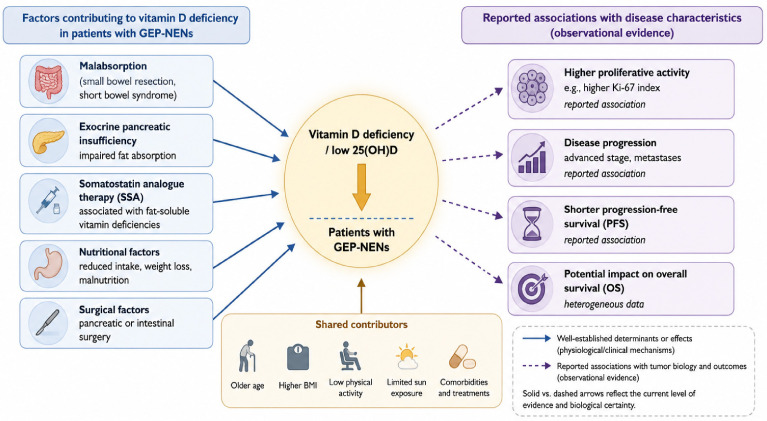
Associations between vitamin D deficiency and selected clinical features in patients with gastroenteropancreatic neuroendocrine neoplasms (GEP-NENs). Left panel: factors potentially contributing to vitamin D deficiency, including malabsorption, exocrine pancreatic insufficiency, nutritional impairment, previous gastrointestinal surgery, and long-term somatostatin analogue (SSA) therapy. Central panel: reduced serum 25-hydroxyvitamin D [25(OH)D] concentrations in patients with GEP-NENs. Right panel: reported associations between low 25(OH)D levels and markers of more aggressive disease, including higher Ki-67 index, disease progression, shorter PFS, and reported associations on OS. Bottom panel: additional factors influencing vitamin D status, such as older age, higher body mass index (BMI), reduced physical activity, limited sunlight exposure, and concomitant comorbidities. Solid arrows indicate clinically recognized determinants of vitamin D deficiency. Dashed arrows represent associations supported mainly by observational and translational studies and should not be interpreted as evidence of causality. Abbreviations: 25(OH)D, 25-hydroxyvitamin D; GEP-NENs, gastroenteropancreatic neuroendocrine neoplasms; SSA, somatostatin analogue; Ki-67, proliferation index; PFS, progression-free survival; OS, overall survival; BMI, body mass index.

Figure 3 summarizes the reported associations between vitamin D deficiency, skeletal complications, and selected markers associated with more aggressive disease behavior in GEP-NENs.

### 4.3. Hereditary Syndromes (MEN1) and Vitamin D-PTH Axis Disorders

Patients with MEN1 represent an important subgroup in the context of vitamin D and calcium-phosphate metabolism disorders. A minority of GEP-NENs occur as part of hereditary syndromes, most commonly MEN1, particularly in pancreatic neuroendocrine tumors [26,56].

MEN1 is a highly penetrant autosomal dominant syndrome caused by germline mutations in the MEN1 tumor suppressor gene encoding menin [26,56,57,58]. Experimental studies suggest that menin may interact with the vitamin D receptor and influence transcriptional pathways involved in bone metabolism and cellular signaling [51]. However, the clinical relevance of these mechanisms in GEP-NENs remains unclear.

Most patients with MEN1 develop primary hyperparathyroidism (PHPT), leading to chronic disturbances in calcium-phosphate homeostasis and dysregulation of the vitamin D-parathyroid hormone axis [26]. Chronic hypercalcemia could complicate the interpretation of vitamin D status and delay recognition of concomitant deficiency. Persistent PTH excess may also contribute to bone loss and impaired bone mineralization, particularly in patients with coexisting vitamin D deficiency [59].

Patients with GEP-NENs could additionally present with reduced bone and muscle mass, even at relatively early disease stages [60]. These findings support long-term monitoring of bone health and nutritional status in this patient population [60,61,62].

### 4.4. General Factors Affecting Vitamin D Status

In addition to disease-specific processes, vitamin D status in patients with GEP-NENs is influenced by several general factors, including age, body mass index (BMI), reduced physical activity, nutritional status, and seasonal sunlight exposure [15]. These factors may further contribute to persistent vitamin D deficiency, particularly in patients with advanced disease or chronic gastrointestinal symptoms.

Because these determinants are not specific to GEP-NENs, they should be interpreted together with disease-related factors such as malabsorption, pancreatic insufficiency, and previous gastrointestinal surgery.

## 5. The Relationship Between Vitamin D Deficiency and Biological Aggressiveness and Prognosis in GEP-NENs

### 5.1. Vitamin D Status and Proliferative Activity of Neuroendocrine Tumors (Ki-67)

In recent years, increasing attention has been paid to the potential relationship between vitamin D status and markers of biological aggressiveness in GEP-NENs, particularly the Ki-67 proliferation index. Ki-67 remains one of the key parameters used for tumor grading, prognostic stratification, and therapeutic decision-making in neuroendocrine neoplasms [2,7,63,64].

Several observational studies suggested that lower 25(OH)D concentrations may be associated with more aggressive disease features. In a study by Altieri et al., vitamin D deficiency was common in patients with GEP-NETs and occurred more frequently in those with higher tumor grade and progressive disease [22]. Albertelli et al. similarly reported significantly higher Ki-67 values in patients with serum 25(OH)D concentrations below 20 ng/mL, while patients with progressive disease had lower baseline vitamin D levels [23].

Taken together, these observations suggest a possible a possible association between low vitamin D status and increased proliferative activity in GEP-NENs. At the same time, the current evidence is derived almost exclusively from observational studies and therefore cannot establish causality [22,23]. In addition, oncological outcomes were not the primary focus of all published studies. Many investigations concentrated instead on the prevalence of vitamin D deficiency, bone health, nutritional status, or supportive metabolic care. For this reason, the available data should be interpreted with caution, and it remains uncertain whether vitamin D status is independently associated with tumor aggressiveness [21,22,23,51,52]. Low vitamin D concentrations may also reflect more advanced disease, nutritional impairment, or chronic illness rather than directly contributing to tumor progression [21,22,23].

Table 4 summarizes the main clinical studies evaluating associations between vitamin D status, tumor proliferation markers, and prognosis in patients with GEP-NENs.

### 5.2. Vitamin D Deficiency and Disease Progression (PFS)

Several studies have evaluated the relationship between vitamin D status and PFS. In the study by Altieri et al., patients with vitamin D deficiency or insufficiency had shorter PFS compared with patients with normal 25(OH)D concentrations [22]. However, interpretation of these findings is limited by the observational design and relatively small study cohort.

Albertelli et al. also observed an association between disease progression and lower baseline 25(OH)D concentrations, but did not demonstrate a significant relationship between vitamin D status and PFS [23]. These differences highlight the heterogeneity of currently available data. At present, the prognostic role of vitamin D in GEP-NENs continues to be uncertain.

### 5.3. Vitamin D Status and Overall Survival (OS), Current Limitations of Evidence

The relationship between vitamin D status and overall survival (OS) in patients with GEP-NENs is even less clear. Massironi et al. reported associations between 25(OH)D concentrations and both PFS and OS. However, multivariate Cox regression analysis did not confirm low 25(OH)D concentration as an independent predictor of mortality [21].

Similarly, Albertelli et al. did not demonstrate a significant association between vitamin D status and overall survival [23]. Taken together, currently available evidence does not support the use of low 25(OH)D concentration as an independent prognostic marker for OS in patients with GEP-NENs.

Vitamin D deficiency could instead reflect overall disease burden, nutritional impairment, chronic inflammation, or more advanced disease phenotype rather than directly influencing survival outcomes [21,22,23].

### 5.4. Summary of Current Clinical Evidence

Available clinical studies suggest that low vitamin D status in patients with GEP-NENs may be associated with markers of increased biological aggressiveness, including higher tumor grade and elevated Ki-67 index. Some studies also reported associations with shorter PFS, whereas data regarding OS remain inconsistent [21,22,23].

Most available data originate from observational studies involving relatively small and heterogeneous patient cohorts. Differences in tumor biology, disease stage, treatment strategies, laboratory methods, and definitions of vitamin D deficiency further limit direct comparisons between studies.

Table 5 summarizes the current interpretation of the available clinical findings and their main limitations. Table 6 complements this overview by summarizing the principal clinical observations together with the current level of supporting evidence.

Overall, the strongest data relate to the high prevalence of vitamin D deficiency and its established metabolic and skeletal consequences. In contrast, associations with tumor aggressiveness and survival outcomes remain based primarily on observational data and should be interpreted cautiously.

Because the Ki-67 proliferation index plays a central role in grading and prognostic stratification of neuroendocrine neoplasms, interpretation of these findings should also consider current WHO, International Agency for Research on Cancer (IARC), and European Neuroendocrine Tumor Society (ENETS) recommendations regarding Ki-67 assessment and tumor classification [2,7,63,64,65,66].

## 6. Indirect Mechanisms Potentially Linking Vitamin D Deficiency with Tumor Aggressiveness in GEP-NENs

Currently available clinical studies do not support a causal relationship between low vitamin D status and more aggressive behavior of GEP-NENs. Several biologically plausible mechanisms have nevertheless been proposed, including modulation of immune responses, chronic inflammation, and immunometabolic pathways that may indirectly influence tumor biology [15].

### 6.1. Vitamin D as a Regulator of Immune Response

Vitamin D contributes to the regulation of both innate and adaptive immune responses through VDR signaling. VDR and vitamin D-metabolizing enzymes are expressed in several immune cell populations, including T lymphocytes, dendritic cells, monocytes, and macrophages [47,48,67]. Experimental studies suggest that active vitamin D metabolites modulate immune responses by reducing Th1 and Th17 responses, decreasing the production of selected pro-inflammatory mediators, and supporting regulatory T-cell function [47,48,67]. These processes could help limit persistent low-grade inflammation. Current evidence, however, is derived almost exclusively from experimental and translational studies, and direct data in patients with GEP-NENs remain limited.

### 6.2. Chronic Inflammation and Neuroendocrine Tumor Progression

Chronic inflammation has been proposed as one of the mechanisms contributing to tumor progression by promoting proliferation, angiogenesis, invasion, metastatic potential, and remodeling of the tumor microenvironment [40,68]. Given the often-indolent course of GEP-NENs, prolonged exposure to inflammatory mediators and metabolic disturbances may influence disease behavior over time. Direct findings supporting these processes in GEP-NENs remain limited [25,26]. Vitamin D deficiency has also been implicated in persistent low-grade inflammation through impaired VDR-dependent regulatory pathways. Evidence supporting this hypothesis comes largely from mechanistic studies and observations in other malignancies and chronic inflammatory disorders [15,69,70,71].

### 6.3. The Vitamin D-Immunometabolism Axis

Immunometabolism describes the interplay between metabolic regulation and immune function within the tumor microenvironment. Experimental studies suggest that vitamin D could influence VDR-dependent pathways involved in cellular differentiation, oxidative stress responses, and inflammatory signaling [32,70,71]. The relevance of these mechanisms to GEP-NEN biology continues to be uncertain. Another concept relevant to vitamin D biology is the “personal vitamin D response index,” which reflects interindividual variability in molecular responses to vitamin D regulation [29]. This variability may partly explain the inconsistent clinical associations reported across studies, although its significance in patients with GEP-NENs has not been established.

### 6.4. Summary of Indirect Mechanisms

Available experimental and translational data support several biologically plausible mechanisms through which vitamin D deficiency could indirectly influence tumor biology, including immune dysregulation, chronic inflammation, and altered immunometabolic signaling [15,40,68,69,71]. Current clinical data, however, do not support a causal relationship between low vitamin D status and disease progression in GEP-NENs. Low serum 25(OH)D concentrations may instead reflect poorer general condition, malnutrition, chronic inflammation, or more advanced disease burden rather than acting as an independent driver of disease progression [69].

## 7. Clinical Consequences of Vitamin D Deficiency in Patients with GEP-NENs

Although the relationship between vitamin D deficiency and tumor aggressiveness in GEP-NENs remains uncertain, the clinical consequences of low vitamin D status extend beyond oncological outcomes alone. The most important effects involve bone metabolism, nutritional status, and overall functional condition.

Several studies have shown that patients with GEP-NENs frequently present with nutritional disturbances and deficiencies of fat-soluble vitamins, including vitamin D [51,72]. These abnormalities may contribute to metabolic complications, impaired bone health, and reduced quality of life.

### 7.1. The Vitamin D-PTH Axis and Bone Health

Vitamin D deficiency contributes to secondary hyperparathyroidism, increased bone turnover, and progressive bone loss. These processes are well established in the development of osteopenia and osteoporosis [73,74,75,76].

In patients with GEP-NENs, the risk of bone metabolism disorders may be further increased by disease-specific factors such as malabsorption, chronic gastrointestinal symptoms, nutritional impairment, and systemic treatment [51,72].

Clinical and review studies suggest that reduced bone mineral density and disturbances in calcium-phosphate metabolism are relatively common in patients with GEP-NENs. For example, reduced bone mineral density was reported in up to 82% of patients with small intestinal neuroendocrine tumors, while vitamin D deficiency was present in approximately 74% of the same cohort [51,73,74]. These abnormalities are likely multifactorial, with vitamin D deficiency representing one component of a broader metabolic phenotype. These complications are clinically relevant because osteoporosis and fracture risk may substantially affect physical function, mobility, and quality of life in patients with chronic disease.

### 7.2. Vitamin D, Muscle Health, and Sarcopenia

In addition to its established role in bone metabolism, vitamin D contributes to muscle function, physical performance, and maintenance of muscle mass. Vitamin D deficiency has been associated with muscle weakness, impaired physical function, and increased risk of sarcopenia in several chronic disease populations.

Patients with GEP-NENs may be particularly vulnerable to loss of muscle mass because of chronic illness, nutritional impairment, malabsorption, reduced physical activity, and treatment-related factors. Emerging findings supports that sarcopenia could adversely affect clinical outcomes and quality of life in patients with neuroendocrine neoplasms, although direct evidence linking vitamin D deficiency to sarcopenia specifically in GEP-NENs remains limited [76].

Assessment of nutritional status, body composition, and muscle strength could complement the evaluation of vitamin D status, particularly in patients with advanced disease, chronic diarrhea, malabsorption, or significant weight loss, providing a more comprehensive evaluation of nutritional and functional status.

### 7.3. Groups of Patients at Increased Risk

Certain subgroups of patients with GEP-NENs appear particularly vulnerable to vitamin D deficiency and its metabolic consequences. This applies especially to patients with chronic malabsorption, malnutrition, previous gastrointestinal surgery, pancreatic insufficiency, or persistent diarrhea [72].

Observational studies reported low 25(OH)D concentrations in approximately two-thirds of patients with GEP-NENs. In a prospective cohort, vitamin D deficiency or insufficiency was identified in 66.8% of patients at baseline, while approximately 15% remained vitamin D deficient despite repeated recommendations for over-the-counter supplementation, suggesting that correction of deficiency may be challenging in selected patients [52].

Patients receiving SSA therapy also require careful nutritional monitoring. Current evidence supports the idea that vitamin D deficiency is more strongly related to malabsorption and overall disease burden than to SSA treatment itself [26].

Patients with MEN1 represent another important high-risk group. In these patients, coexisting primary hyperparathyroidism may lead to chronic disturbances in calcium-phosphate metabolism, while hypercalcemia could complicate interpretation of vitamin D status and delay recognition of deficiency [15]. Long-term disturbances in the vitamin D-parathyroid hormone axis are thought to additionally increase the risk of skeletal complications.

### 7.4. Practical Implications in Clinical Care

Given the high prevalence of vitamin D deficiency in patients with GEP-NENs, assessment of serum 25(OH)D concentration should be considered particularly in patients at increased risk of deficiency. This includes patients with malabsorption, previous gastrointestinal or pancreatic surgery, chronic diarrhea, malnutrition, osteopenia, osteoporosis, or MEN1-associated primary hyperparathyroidism [51,72].

Although observational studies have reported associations between vitamin D status and oncological outcomes, including PFS and OS, current data do not demonstrate that correction of vitamin D deficiency directly improves these outcomes in patients with GEP-NENs [21,22,23,52].

In routine clinical practice, correction of vitamin D deficiency remains an important component of metabolic and skeletal care [74,75,77].

However, the role of vitamin D supplementation in the primary prevention of osteoporosis and fragility fractures continues to be controversial. While correction of vitamin D deficiency is recommended as part of comprehensive osteoporosis management, the recent Endocrine Society Clinical Practice Guideline concluded that current evidence is insufficient to support universal vitamin D supplementation or fixed serum 25(OH)D target concentrations for disease or fracture prevention in the general population [74,75,77].

Taken together, these findings support identification and treatment of vitamin D deficiency according to current bone and metabolic health recommendations while taking into account the specific clinical characteristics of patients with GEP-NENs.

## 8. Therapeutic Interventions: Supplementation and Vitamin D Analogues

### 8.1. Vitamin D Supplementation in Patients with GEP-NENs

The high prevalence of vitamin D deficiency in patients with GEP-NENs supports consideration of supplementation as part of routine metabolic and bone health care [51].

Currently available evidence regarding supplementation in GEP-NENs remains limited and is based mainly on observational studies. Robbins et al. reported that simple recommendations regarding over-the-counter vitamin D supplementation were associated with improvements in serum 25(OH)D concentrations in patients with GEP-NETs [52].

At the same time, some patients remained vitamin D-deficient despite supplementation. This suggests that selected individuals, particularly those with malabsorption or previous gastrointestinal surgery, may require more individualized management strategies [52].

In the same study, previous abdominal surgery was associated with lower 25(OH)D concentrations, whereas SSA therapy was not identified as an independent predictor of vitamin D status in multivariable analysis [52].

Altieri et al. additionally emphasized the increased risk of osteopenia and osteoporosis in patients with GEP-NETs and highlighted the importance of identifying and correcting vitamin D deficiency as part of supportive metabolic care [51].

Importantly, there are currently no randomized clinical trials evaluating the effect of vitamin D supplementation on oncological outcomes such as PFS or OS in patients with GEP-NENs. For this reason, vitamin D supplementation should currently be considered a supportive metabolic intervention rather than an anticancer treatment strategy [78].

### 8.2. Safety of Supplementation and Practical Considerations

Vitamin D supplementation is generally considered safe when administered according to current clinical recommendations and with appropriate monitoring of calcium-phosphate metabolism in selected patients.

Particular caution could be required in patients with MEN1-associated primary hyperparathyroidism. In these patients, chronic disturbances of the calcium-parathyroid hormone axis may increase the risk of hypercalcemia during supplementation [26]. Current MEN1 management guidelines emphasize the importance of monitoring calcium-phosphate metabolism and long-term bone health in this patient population [26].

In clinical practice, periodic assessment of serum 25(OH)D concentration should be considered, particularly in patients at increased risk of persistent deficiency. The frequency of monitoring should be individualized according to baseline vitamin D status, nutritional status, the presence of malabsorption, previous gastrointestinal surgery, pancreatic insufficiency, and response to supplementation. This includes patients with previous gastrointestinal surgery, chronic malabsorption, pancreatic insufficiency, malnutrition, or chronic diarrhea [72,76].

Depending on the clinical setting, monitoring of calcium and PTH concentrations may also be appropriate, particularly in patients with suspected calcium-phosphate metabolism disorders.

### 8.3. Vitamin D Analogues: Preclinical Evidence and Future Perspectives

In addition to standard supplementation, several synthetic vitamin D analogues and VDR agonists have been investigated in experimental oncology models. These compounds were developed to preserve the antiproliferative properties of calcitriol while reducing the risk of hypercalcemia.

Experimental studies indicate that VDR-dependent signaling may influence pathways involved in cell proliferation, differentiation, apoptosis, and inflammatory regulation [70]. Although these findings encouraged the development of vitamin D analogues, their clinical application has remained limited. Several low-calcemic analogues, including EB1089, inecalcitol, and paricalcitol, have been evaluated in early clinical studies. While acceptable safety profiles have generally been reported, consistent evidence of clinical benefit is still lacking [77]. Similarly, other reviews concluded that promising preclinical activity has not yet translated into routine oncology practice because of limitations related to calcium metabolism, dosing strategies, and clinical trial design [79,80,81].

The available findings on vitamin D supplementation and vitamin D analogues are summarized in Table 7.

Importantly, no clinical studies have demonstrated the efficacy of vitamin D analogues specifically in patients with GEP-NETs. Their role in this setting should therefore still be regarded as experimental [70,77,79].

### 8.4. Practical Considerations for Vitamin D Assessment and Supplementation

Although disease-specific recommendations for vitamin D supplementation in patients with GEP-NENs are currently unavailable, several practical considerations can be derived from the available evidence and relevant endocrine and oncology guidelines. These considerations mainly concern the identification of patients at increased risk of vitamin D deficiency, appropriate monitoring, and correction of deficiency as part of supportive metabolic care rather than anticancer treatment. The main practical considerations are summarized in Table 8.

The practical considerations presented in this table are based on the currently available findings and relevant endocrine and oncology guidelines. They should not be interpreted as GEP-NEN-specific clinical practice guidelines.

## 9. Limitations of Current Evidence

Despite increasing interest in the role of vitamin D in neuroendocrine neoplasms, currently available clinical data continues to be limited and should be interpreted cautiously.

Most published studies are observational and retrospective. This substantially limits the ability to establish causal relationships between vitamin D deficiency and tumor behavior [21,22,23]. It therefore remains unclear whether low 25(OH)D concentration contributes to disease progression or instead reflects more advanced disease, malnutrition, chronic inflammation, or poorer overall clinical condition [21,22,23,69].

Another relevant limitation is the marked heterogeneity of GEP-NENs. Differences in primary tumor location, histological differentiation, Ki-67 proliferation index, disease stage, and treatment strategies make direct comparison between studies difficult and limit generalizability of the available findings [7,63].

Interpretation of the literature is further complicated by methodological differences between studies. These include variability in definitions of vitamin D deficiency, different cut-off values for 25(OH)D concentration, and differences in laboratory assessment methods [21,22,23,69].

Many studies also lacked comprehensive adjustment for potential confounding factors such as tumor burden, malabsorption, nutritional status, systemic treatment, and concomitant metabolic disorders. For this reason, currently available evidence should still be regarded primarily as hypothesis-generating rather than definitive.

## 10. Integrated Monitoring and Personalized Medicine

Due to the dual role of vitamin D in bone metabolism and broader metabolic regulation, assessment of vitamin D status may be considered part of integrated multidisciplinary care in patients with GEP-NENs, particularly in the context of bone health, nutritional status, and long-term metabolic complications.

Periodic assessment of serum 25(OH)D concentration could be particularly relevant at diagnosis and during long-term follow-up in patients at increased risk of deficiency. However, the optimal serum 25(OH)D concentration continues to be uncertain. Some regional metabolic and osteoporosis-oriented recommendations have proposed serum 25(OH)D concentrations of 30–50 ng/mL as optimal. In contrast, the 2024 Endocrine Society Clinical Practice Guideline found insufficient evidence to define universal outcome-specific target concentrations and no longer endorses the previous target of 30 ng/mL for the general population [77]. Importantly, these recent recommendations were developed primarily for individuals without established indications for vitamin D treatment or testing and should therefore be applied cautiously to patients with GEP-NENs and disease-related risk factors for deficiency. Beyond its established contribution in calcium-phosphate homeostasis and bone metabolism, vitamin D has also attracted interest as a potential marker reflecting broader interactions between tumor biology, inflammation, nutritional status, and host metabolic condition.

Several observational studies reported associations between low 25(OH)D concentrations and higher Ki-67 proliferation index values, disease progression, and markers of more advanced disease phenotype [15,21,22,23]. However, these findings should be interpreted cautiously because currently available findings remain observational and do not support causal conclusions.

Future translational research may integrate vitamin D status into broader multidimensional models of tumor biology, inflammation, nutritional status, and metabolic health in patients with GEP-NENs [7,63,68].

**Figure 4:**
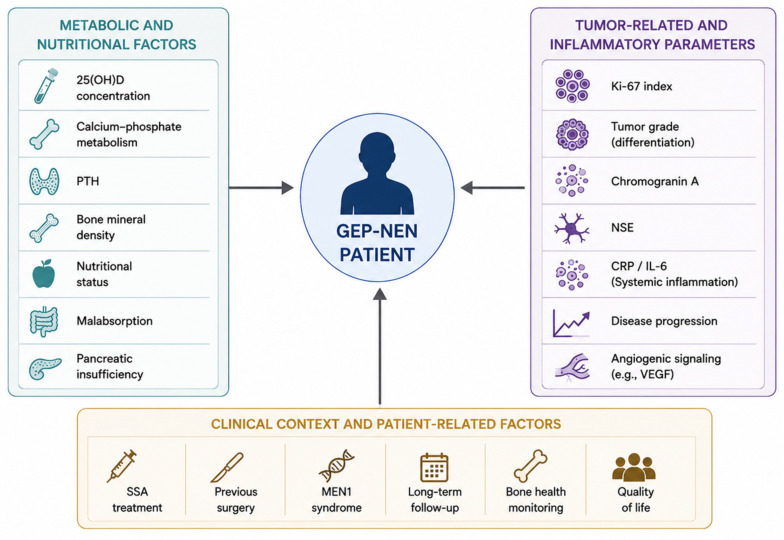
Conceptual framework integrating vitamin D status with selected metabolic, nutritional, inflammatory, and tumor-related parameters in patients with gastroenteropancreatic neuroendocrine neoplasms (GEP-NENs). The scheme summarizes factors that may influence clinical assessment and supportive management, including bone metabolism, nutritional status, inflammatory markers, and tumor-related characteristics. It is presented as a conceptual framework to guide future translational research and should not be interpreted as evidence of causal relationships. Abbreviations: 25(OH)D, 25-hydroxyvitamin D; GEP-NENs, gastroenteropancreatic neuroendocrine neoplasms; PTH, parathyroid hormone; NSE, neuron-specific enolase; CRP, C-reactive protein; IL-6, interleukin 6; SSA, somatostatin analogue; VEGF, vascular endothelial growth factor.

Figure 4 presents a conceptual framework illustrating how vitamin D status could be integrated with selected clinical, inflammatory, metabolic, and tumor-related parameters in future translational research on GEP-NENs.

In summary, vitamin D deficiency is common and clinically relevant in patients with GEP-NENs. Although current data do not support vitamin D supplementation as an anticancer strategy, assessment and correction of deficiency remain important because of their established metabolic and skeletal consequences.

In the future, vitamin D status could become part of broader monitoring strategies integrating metabolic, inflammatory, nutritional, and tumor-related parameters. However, the clinical relevance of such approaches remains uncertain and requires confirmation in prospective studies before implementation in routine clinical practice.

Recent expert statements and contemporary guidance papers increasingly highlight the importance of nutritional status, metabolic complications, and bone health in patients with neuroendocrine neoplasms, particularly in those with chronic diarrhea, malabsorption, bowel resection, pancreatic insufficiency, MEN1-associated disease, or long-term somatostatin analogue therapy [85,86,87,88,89,90]. Current ENETS guidance documents emphasize regular nutritional assessment and supportive metabolic care in high-risk patients [85,86,87,88,89,90].

In this setting, evaluation of vitamin D status may be particularly relevant in selected patients at increased risk of deficiency and skeletal complications.

## 11. Future Directions

Vitamin D deficiency is frequently encountered in patients with GEP-NENs and most likely reflects the combined effects of disease-related factors, including malabsorption, nutritional disturbances, chronic illness, metabolic dysfunction, and previous gastrointestinal surgery [15,21,22,23]. Several observational studies have linked low 25(OH)D concentrations with markers of more aggressive disease, including higher Ki-67 proliferation index values and shorter progression-free survival. However, these observations do not establish a causal relationship between vitamin D deficiency and tumor progression [21,22,23].

Despite growing interest in this field, several clinically relevant questions remain unresolved. Well-designed prospective studies are needed to better define the clinical significance of vitamin D status in patients with GEP-NENs. Standardized definitions of vitamin D deficiency together with harmonized laboratory methods would improve comparability across future studies. Careful patient stratification according to primary tumor site, histological grade, Ki-67 proliferation index, MEN1 status, nutritional status, previous gastrointestinal surgery, and systemic treatment exposure will also be essential [7,52,63].

Clarifying the role of VDR signaling, vitamin D-metabolizing enzymes, inflammation, immunometabolism, and the tumor microenvironment may improve our understanding of vitamin D biology in GEP-NENs [15,70]. Randomized clinical trials are also required to determine whether correction of vitamin D deficiency improves clinically relevant outcomes, particularly bone health, quality of life, metabolic complications, and other supportive care endpoints [52,79].

## 12. Conclusions

At present, assessment of vitamin D status should be regarded primarily as part of supportive metabolic care rather than a strategy for improving oncological outcomes. Given the high prevalence of deficiency and its well-established skeletal consequences, identification and correction of vitamin D deficiency remain clinically justified in patients with GEP-NENs [51,72,79].

Experimental and translational studies continue to improve our understanding of the relationship between vitamin D signaling, inflammation, immune regulation, and tumor biology. Nevertheless, these observations have not yet translated into convincing clinical evidence, and vitamin D supplementation should not currently be considered an anticancer treatment strategy. Current ENETS and European Society for Medical Oncology (ESMO) recommendations increasingly emphasize nutritional and metabolic assessment as integral components of comprehensive care for patients with neuroendocrine neoplasms. Within this framework, evaluation of vitamin D status may be particularly relevant in selected high-risk patients [86,87,88,89,90].

Based on the data currently available, vitamin D in GEP-NENs should primarily be regarded as a marker of nutritional and metabolic status rather than a validated therapeutic target. Whether vitamin D signaling independently influences tumor biology remains uncertain and should be addressed in future prospective clinical and translational studies [91].

## Figures and Tables

**Table 2:** Main mechanisms of vitamin D receptor signaling discussed in cancer biology.

Level of Action	Main Mechanism	Potential Biological Relevance	References
Genomic	VDR-RXR complex regulates transcription of target genes	Regulation of cell cycle, apoptosis, differentiation, angiogenesis, and inflammatory pathways	[17,29,30,39]
Epigenetic	Modulation of chromatin accessibility and interactions with transcriptional regulators	Context-dependent regulation of transcriptional activity and cellular response	[43,44,45]
Non-genomic	Rapid signaling responses involving secondary messengers and membrane-associated pathways	Potential effects on cell migration, adhesion, survival, and short-term cellular responses	[41,42,46]

Abbreviations: VDR, vitamin D receptor; RXR, retinoid X receptor.

**Table 3:** Major vitamin D metabolic pathways and their proposed relevance in cancer biology.

Metabolic Pathway	Key Enzymes	Key Metabolites	Main Points of Regulation	Proposed Relevance in Cancer Biology	References
Classic hepatic-renal pathway	CYP2R1, CYP27B1	25(OH)D, 1,25(OH)_2_D	25(OH)D, 1,25(OH)2D	Regulation of pathways involved in proliferation, differentiation, and apoptosis	[29,30,32]
Catabolism and signal inactivation	CYP24A1	24-hydroxylated metabolites	Induction by 1,25(OH)2D, phosphates, FGF-23	Reduced VDR signaling and altered local vitamin D activity	[31,32]
Extra-renal local activation	CYP27B1 (tissue expression)	Locally produced 1,25(OH)_2_D	Immunological and inflammatory signals (e.g., cytokines)	Modulation of immune and inflammatory responses within the tumor microenvironment	[33,34,35]
Non-canonical CYP11A1-dependent pathway	CYP11A1	20(OH)D, 20,23(OH)_2_D and other related metabolites	Dependent on tissue, local steroidogenic activity	Biological activity with potentially lower calcemic effects in preclinical models	[36,37]
Interactions with ROR receptors	RORα, RORγ	Non-canonical metabolites	Ligand-dependent and cell-specific regulation	Possible involvement in inflammatory and immune-related signaling pathways including Th17/IL-17 axis	[36,37,38,46,49]

Abbreviations: 25(OH)D, 25-hydroxyvitamin D; 1,25(OH)_2_D, 1,25-dihydroxyvitamin D; VDR, vitamin D receptor; FGF-23, fibroblast growth factor 23; ROR, retinoic acid-related orphan receptor; IL-17, interleukin 17; Th17, T helper 17 lymphocytes.

**Table 4:** Clinical evidence evaluating vitamin D status, tumor aggressiveness, and clinical outcomes in patients with GEP-NENs and mixed NEN cohorts.

Author (Year)	Population (*n*)	Main Findings Regarding Vitamin D Status	Associations with Ki-67/Grade	PFS Findings	OS Findings	Main Limitations
Massironi et al. (2017) [21]	GEP-NEN cohort (*n* = 138)	High prevalence of reduced 25(OH)D concentrations	No clear independent association confirmed	Lower 25(OH)D associated with shorter PFS in univariate analyses	No independent association with OS in multivariate analysis	Observational design, heterogeneous cohort, limited sample size
Altieri et al. (2022) [22]	Mixed NET/NEN cohort (*n* = 75)	Vitamin D deficiency common in the study population	Lower 25(OH)D associated with higher tumor grade and more aggressive disease features	Shorter PFS observed in patients with vitamin D deficiency/insufficiency	OS data limited	Observational study, limited cohort size, possible confounding factors
Albertelli et al. (2023) [23]	Mixed NET cohort (*n* = 172)	Lower baseline 25(OH)D concentrations in patients with disease progression	Higher Ki-67 values in patients with 25(OH)D < 20 ng/mL	No statistically significant association between 25(OH)D and PFS	No significant association with OS	Retrospective design, heterogeneous population, limited statistical power

Abbreviations: 25(OH)D, 25-hydroxyvitamin D; GEP-NENs, gastroenteropancreatic neuroendocrine neoplasms; Ki-67, proliferation index; PFS, progression-free survival; OS, overall survival.

**Table 5:** Clinical and mechanistic factors potentially contributing to vitamin D deficiency and related metabolic consequences in GEP-NENs.

Clinical or Biological Factor	Proposed Relevance in GEP-NENs	Type of Evidence	Main Limitations
VDR-dependent signaling	Potential involvement in proliferation, apoptosis, and immune regulation	Experimental and translational studies [29,30,32,39]	Predominantly preclinical evidence
ROR-related pathways and inflammation	Possible modulation of inflammatory and immune signaling	Experimental studies [36,37,38,46,49]	Limited direct validation in GEP-NEN models
Intestinal malabsorption	Reduced absorption of fat-soluble vitamins, including vitamin D	Clinical observational studies [50,51,52,53]	Heterogeneous patient populations and surgical history
Exocrine pancreatic insufficiency	Chronic malabsorption and impaired vitamin D absorption	Clinical and nutritional studies [51,53]	Often coexists with advanced disease and prior surgery
Somatostatin analogue (SSA) therapy	Possible indirect contribution to fat-soluble vitamin deficiencies	Observational studies [52,54,55]	Difficult to separate treatment effects from disease-related factors
Nutritional impairment and weight loss	Reduced dietary intake and altered nutritional status	Observational clinical data [50,51,53]	Frequently associated with advanced disease burden
MEN1 and primary hyperparathyroidism	Dysregulation of calcium-phosphate metabolism and PTH-vitamin D axis	Clinical and guideline-based evidence [26,56,57,58,59]	Limited direct evidence regarding vitamin D outcomes in MEN1-associated GEP-NENs
Bone metabolism disturbances	Increased risk of bone loss and impaired mineralization	Clinical studies and supportive metabolic evidence [59,60]	Limited prospective bone-health studies in GEP-NEN populations

Abbreviations: GEP-NENs, gastroenteropancreatic neuroendocrine neoplasms; SSA, somatostatin analogue; MEN1, multiple endocrine neoplasia type 1; PTH, parathyroid hormone; VDR, vitamin D receptor; ROR, retinoic acid-related orphan receptor.

**Table 6:** Strength of current evidence regarding vitamin D status and clinical outcomes in GEP-NENs.

Clinical Issue	Current Evidence	Strength of Evidence	Clinical Interpretation
Prevalence of vitamin D deficiency	Consistently reported across observational studies	Moderate	Vitamin D deficiency appears common in GEP-NENs
Association with Ki-67 and tumor grade	Reported in several cohorts	Low	Possible association with more aggressive disease
Association with progression-free survival	Inconsistent findings	Low	Prognostic value remains uncertain
Association with overall survival	Largely negative or inconsistent findings	Very low	Not currently supported as an independent prognostic marker
Effects of vitamin D supplementation on oncological outcomes	No randomized evidence	Very low	No evidence supporting anticancer benefit
Bone health and metabolic consequences	Supported by broader clinical evidence	Moderate	Clinically relevant indication for assessment and supplementation

Abbreviations: GEP-NENs, gastroenteropancreatic neuroendocrine neoplasms; Ki-67, Ki-67 proliferation index.

**Table 7:** Available evidence on vitamin D supplementation and vitamin D analogues relevant to GEP-NETs and oncology.

Reference	Publication Type	Intervention/Focus	Main Findings	Clinical Implications
Altieri et al. [51]	Narrative review	Bone health in GEP-NETs; role of vitamin D and MEN1	Bone metabolism in patients with GEP-NETs may be influenced by vitamin D deficiency, hormonal hypersecretion, nutritional status, previous surgery, bone metastases, and MEN1-associated bone disease. The review also points to the lack of specific recommendations for bone health screening in this population.	Highlights the need for routine assessment of bone health and vitamin D status in patients with GEP-NETs. Anticancer effects of vitamin D supplementation were not addressed.
Robbins et al. [52]	Prospective observational study; 183 patients with GEP-NETs; 24-month follow-up	Advice to use over-the-counter cholecalciferol (1000–2000 IU/day)	Serum 25(OH)D concentrations increased during follow-up and vitamin D deficiency became less frequent. Despite supplementation, approximately 15% of patients remained deficient. Previous abdominal surgery, but not SSA therapy, was associated with lower 25(OH)D concentrations.	Routine supplementation improved vitamin D status in many patients, although additional supplementation may be required in selected individuals. Effects on PFS and OS were not evaluated.
Duffy et al. [81]	Review	Vitamin D receptor signaling and vitamin D analogues in oncology	Calcitriol and several synthetic vitamin D analogues inhibited tumor growth in experimental models. Low-calcemic analogues, including EB1089, inecalcitol, and paricalcitol, were evaluated in early clinical studies and showed acceptable safety, although convincing anticancer efficacy has yet to be demonstrated. Some studies also suggested improved responses to standard anticancer therapies when used as an adjunct.	Early clinical studies reported acceptable safety, although convincing clinical benefit has not been demonstrated.
Leyssens et al. [82]	Review	Vitamin D analogues in cancer	Experimental studies identified vitamin D analogues with antiproliferative activity and reduced calcemic effects compared with calcitriol. Despite encouraging preclinical findings, none has been introduced into routine cancer treatment.	No vitamin D analogue is currently used in routine oncology practice.
Masuda and Jones [83]	Review	Anticancer potential of calcitriol and vitamin D analogues	Calcitriol and its analogues regulate pathways involved in cell proliferation, apoptosis, angiogenesis, and metastatic spread. Experimental studies yielded encouraging results and prompted clinical evaluation of different dosing schedules and combination strategies.	Routine clinical application has remained challenging despite encouraging experimental findings.
Trump [84]	Review	Vitamin D signaling and anticancer therapy	Calcitriol and other vitamin D compounds exhibited multiple anticancer effects in preclinical models, including inhibition of proliferation, migration, and angiogenesis. Clinical studies have been difficult to interpret because of limitations in trial design and drug development.	Methodological limitations continue to hamper interpretation of the available clinical evidence.

Abbreviations: 25(OH)D, 25-hydroxyvitamin D; GEP-NETs, gastroenteropancreatic neuroendocrine tumors; MEN1, multiple endocrine neoplasia type 1; OS, overall survival; PFS, progression-free survival; SSA, somatostatin analogue.

**Table 8:** Practical considerations for vitamin D assessment and supplementation in patients with GEP-NENs.

Clinical Scenario	Practical Consideration	Supporting Evidence
Newly diagnosed GEP-NENs	Consider measuring serum 25(OH)D concentration at baseline, given the high prevalence of vitamin D deficiency reported in patients with GEP-NENs.	[21,22,23]
Previous gastrointestinal surgery, malabsorption, or pancreatic insufficiency	These conditions are associated with an increased risk of vitamin D deficiency and may justify closer monitoring of vitamin D status.	[22,51,52]
Long-term follow-up	Reassess vitamin D status according to the clinical course and individual risk factors for persistent deficiency.	[21,22,23,51,52]
Increased fracture risk or osteoporosis	Consider assessment of calcium, phosphate, PTH, and bone mineral density when clinically indicated, followed by management according to current osteoporosis recommendations.	[51,79]
Vitamin D deficiency	Correct vitamin D deficiency according to current endocrine recommendations. The primary aim of supplementation is to support skeletal and metabolic health rather than improve oncological outcomes.	[51,79]
Vitamin D analogues	Vitamin D analogues remain investigational, and current evidence does not support their routine clinical use.	[81,82,83,84]

Abbreviations: 25(OH)D, 25-hydroxyvitamin D; GEP-NENs, gastroenteropancreatic neuroendocrine neoplasms; PTH, parathyroid hormone.

## Data Availability

No new data were created or analyzed in this study. Data sharing is not applicable to this article.
